# Tracing IgE-Producing Cells in Allergic Patients

**DOI:** 10.3390/cells8090994

**Published:** 2019-08-28

**Authors:** Julia Eckl-Dorna, Sergio Villazala-Merino, Nicholas James Campion, Maria Byazrova, Alexander Filatov, Dmitry Kudlay, Antonina Karsonova, Ksenja Riabova, Musa Khaitov, Alexander Karaulov, Verena Niederberger-Leppin, Rudolf Valenta

**Affiliations:** 1 Department of Otorhinolaryngology, Medical University of Vienna, Vienna A-1090, Austria; 2 NRC Institute of Immunology FMBA of Russia, Moscow 115478, Russia; 3 Department of Clinical Immunology and Allergy, Sechenov First Moscow State Medical University, Moscow 119435, Russia; 4 Division of Immunopathology, Department of Pathophysiology and Allergy Research, Center for Pathophysiology, Infectiology and Immunology, Medical University of Vienna, Vienna A-1090, Austria

**Keywords:** allergy, IgE, human, mouse, CD23, FcεRI, B cell, T cell, tracing, targeting, therapy

## Abstract

Immunoglobulin E (IgE) is the key immunoglobulin in the pathogenesis of IgE associated allergic diseases affecting 30% of the world population. Recent data suggest that allergen-specific IgE levels in serum of allergic patients are sustained by two different mechanisms: inducible IgE production through allergen exposure, and continuous IgE production occurring even in the absence of allergen stimulus that maintains IgE levels. This assumption is supported by two observations. First, allergen exposure induces transient increases of systemic IgE production. Second, reduction in IgE levels upon depletion of IgE from the blood of allergic patients using immunoapheresis is only temporary and IgE levels quickly return to pre-treatment levels even in the absence of allergen exposure. Though IgE production has been observed in the peripheral blood and locally in various human tissues (e.g., nose, lung, spleen, bone marrow), the origin and main sites of IgE production in humans remain unknown. Furthermore, IgE-producing cells in humans have yet to be fully characterized. Capturing IgE-producing cells is challenging not only because current staining technologies are inadequate, but also because the cells are rare, they are difficult to discriminate from cells bearing IgE bound to IgE-receptors, and plasma cells express little IgE on their surface. However, due to the central role in mediating both the early and late phases of allergy, free IgE, IgE-bearing effector cells and IgE-producing cells are important therapeutic targets. Here, we discuss current knowledge and unanswered questions regarding IgE production in allergic patients as well as possible therapeutic approaches targeting IgE.

## 1. Introduction

Immunoglobulin E (IgE) associated allergic diseases in their various forms affect approximately 30% of the world population. Symptoms may range from relatively mild, such as rhinoconjunctivitis, to potentially life-threatening, such as asthma or anaphylaxis. Development of allergic disease is associated both with environmental and individual genetic factors [1]. The latter includes a genetic predisposition towards allergen-specific immune responses and factors promoting Th2 responses as well as IgE production [1,2,3]. IgE is a key player in development and maintenance of allergic disease. Symptoms of the early phase of allergic inflammation are driven by mediators released from basophils and mast cells upon allergen-induced crosslinking of IgE bound to its high affinity surface receptor (FcεRI) [4,5]. Furthermore, IgE also contributes to the enhancement of the late phase response. IgE is present on the surface of antigen presenting cells (APCs) bound to FcεRI or the low affinity IgE receptor CD23. Allergen-IgE complexes are internalized by these receptor on APCs and presented via major histocompatibility complex II (MHCII), thus augmenting allergen-specific T cell responses [6,7,8,9]. IgE may also bind to soluble IgE receptors and IgE-binding proteins, e.g., soluble CD23 or epsilon binding protein [10].

Among all immunoglobulin subclasses, IgE stands out with respect to function, half-life, and low serum concentration. With a serum concentration of 5 × 10^−5^ mg/mL [11] it represents only 0.0005% of total free serum Igs in non-atopic adults [12]. Its half-life of 2 days within the serum is rather short compared to the half-life of, for example, IgG_1_, lasting 21 days [11]. In contrast to many other subclasses, IgE does not activate complement but exerts its role through binding to its cognate receptors [11]. It has been shown that long-lasting removal of IgE from the circulation is difficult to achieve. After extracorporeal immunoadsorption, IgE levels return to baseline within a week after treatment, even in the absence of exogenous allergen stimulus [13]. This shows that despite its low abundance, IgE is continuously produced to maintain constant IgE levels in blood.

The central role of IgE in mediating allergic diseases makes it an important and attractive target for development of novel therapeutic approaches [14,15,16]. So far, only the anti-IgE antibody omalizumab has been marketed and successfully reduces the burden of severe and otherwise uncontrollable asthma [17,18,19]. Several new approaches such as depleting IgE through extracorporal IgE immunoabsorption [13,20] as well as specifically targeting effector [21,22] or IgE+ B cells [23] are being explored and will be discussed in this review.

## 2. IgE Production

The pathway of B cell differentiation with respect to the nature and location of potential IgE+ memory cells as well as long-lived IgE producing plasma cells in allergy is still not completely understood. Investigation of human IgE responses are impeded by the fact that IgE-producing cells and B cells are rare in human tissues that are easy to access, such as blood, nasal mucosa, or tonsils [24,25,26,27]. Therefore, most of our current knowledge on the mechanisms underlying IgE production is based on data from murine models. Advances in this field have been made in the past decade due to the generation of fluorescent protein reporter IgE mice [28]. Experiments in these mice led to the perception that IgE expressing B cells only transiently contribute to the germinal center reaction and are rather biased towards a plasma cell fate [29,30].

### 2.1. Murine Models to Investigate IgE-Production and Allergic Disease

Murine models are very valuable for our understanding of general mechanisms of allergy in humans as well as for testing novel therapeutic approaches, but it is necessary to bear several differences in mind (Table 1). The cytokine milieu required for induction of class switch to IgE differs between mice and humans. While IL-4 alone directs class switching to IgE and IgG_1_ in mice [31], both IL-4 and IL-13 contribute to IgE synthesis in humans [32]. In addition, mice do not mount IgG_4_ subclass responses like allergic patients. With regard to allergen epitope specific Ig responses, the two species differ. Recent studies have indicated that the allergen-specific IgE responses in mice develop by switching from IgG_1_ to IgE [29]. In this respect, it has been shown that IgG raised in mice towards the major grass pollen allergens Phl p 1, 2, 5, or dog albumin were able to block IgE binding, which indicates that, at least for these selected allergens, IgG and IgE of sensitized mice recognize the same epitope [33]. However, direct class switching from IgM to IgE has also been observed in mice [34]. In contrast, IgE and IgG in humans have been shown to recognize distinct epitopes of the allergen [35,36]. This is also supported by the fact that only IgE but no other immunoglobulin subclass is boosted upon seasonal allergen exposure [37,38].

With respect to the composition of the immune system, there are further differences to consider. While lymphocytes are the predominant mononuclear cells present in the peripheral blood of mice (representing 75–90% of total leukocytes [39]), neutrophils (50–70%) are predominant in humans with lymphocytes comprising only 30–50% of all leukocytes [40].

Another caveat is that mouse models mimicking allergic rhinitis, a key feature of human disease, are rare and are hampered by technical difficulties such as measuring isolated nasal obstruction. Additionally, the nasal anatomy of mice differs from human anatomy [54,55]. In allergic asthma, murine models have provided important insights into immunologic mechanisms. However, several shortcomings are clearly visible: Mice are inbred and live in specific pathogen-free facilities, whereas humans bear individual genetic backgrounds and are continuously exposed to a myriad of environmental agents, many of them known to influence and modulate airway inflammation [56,57]. Human airways are, for instance, richer in submucosal glands and show a greater variety of airway structure [56]. In terms of pathophysiological mechanisms, there are marked differences in eosinophil degranulation and infiltration as well as in the effects of mast cell degranulation on bronchoconstriction between mice and humans [57]. In fact, murine models of asthma sometimes resemble features of Type IV hypersensitivity rather than Type I allergy [3,58,59]. To summarize, there are some important differences between murine and human allergic responses that need to be taken into account when interpreting murine data.

### 2.2. Human IgE Production

**Figure 1:**
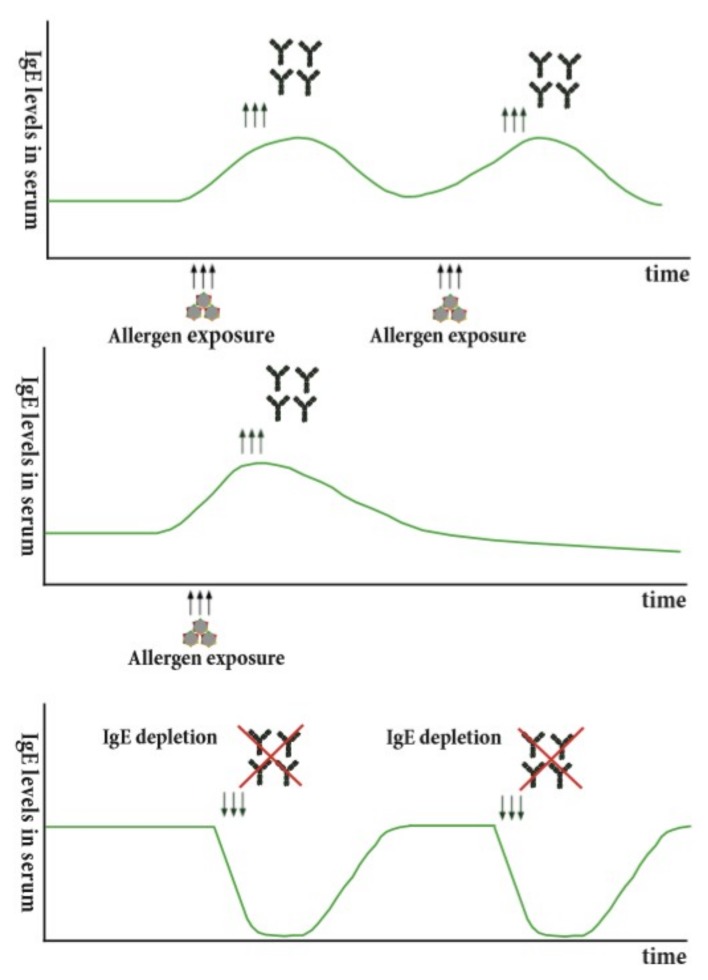
Potential mechanisms for maintenance of continuous IgE production. (Top) Repeated allergen exposure maintains IgE production. (Middle) Lack of allergenic stimulation leads to a slow steady decline in IgE production. (Bottom) Depletion of IgE, e.g., using IgE immune adsorption, leads only to a temporary decline of IgE levels, which subsequently return to baseline levels.

In humans, allergen-specific IgE memory persists over years or even decades, even in the absence of antigenic stimulation [60]. At the same time, seasonal allergen exposure can induce a rapid increase of allergen-specific IgE levels and boosts IgE levels [37,61]. These findings indicate that there may be two different processes governing IgE production: one that continuously replenishes the IgE pool—perhaps long-lived plasma cells [62,63]—and another that is inducible upon allergen contact. In fact, there are various studies in favor of this hypothesis. Firstly, the observation that the reduction in IgE levels after depletion of IgE from human blood using immune apheresis is only temporary and returns to pre-treatment levels within a week in the absence of antigenic stimulus [13,20] (Figure 1, Bottom) indicates the presence of continuous IgE production. The mechanism how this IgE production is maintained is not fully understood. It is conceivable that repeated boosts of allergen-specific IgE production are required to keep this IgE production ongoing (Figure 1, Top). Activation could either be achieved by repeated allergen-contact (e.g., seasonal exposure) or in the absence of this also by polyclonal activation (e.g., microbial stimulation, bystander T-help) as has been described for the maintenance of antigen-specific IgG memory [64]. If repeated activation sustains IgE levels and the specificities of IgE, a lack of antigenic exposure should lead to a slow decline in IgE production (Figure 1, Middle). This could be investigated in the context of allergy by relocation of an allergic patient to an allergen-free area for seasonal allergens or dietary restriction for food derived allergens. In support of this, it has been observed that lack of antigen exposure in the context of egg or cow-milk allergy leads to a decline in allergen-specific IgE levels [65,66,67].

Aside from continuous IgE production there appears to be a second mechanism where IgE production is boosted upon allergen contact as seasonal allergen exposure via the respiratory mucosa has been shown to induce a strong increase of allergen-specific IgE levels [37,61]. A rise in allergen-specific T cell proliferation has also been observed during the season [68], but within individual patients B and T cell responses are poorly associated [69]. In humans the origin of IgE-switched B cells is not yet entirely clear [52] with studies both in favor of direct and sequential class switching. The observation that upon seasonal allergen exposure only established allergen-specific memory is boosted [37] supports the hypothesis of direct class switching and suggests the existence of IgE+ memory B cells that can be stimulated upon allergen contact. Furthermore, no de novo sensitizations [70] or changes in allergen-specific IgM, IgG, or IgA production [37,38] have been described upon allergen challenge in humans.

However, there is also evidence indicating that sequential class switching occurs in humans [46,47,48,49,50,51,52]. In this respect, the presence of sγ switch circles was observed in supernatant of nasal mucosa explants [47]. Furthermore, IgH repertoire analysis in blood in allergic patients showed that IgE cells may also derive from secondary isotype switching of mutated IgG_1_ expressing cells [46,52]. In this line, treatment with the human monoclonal antibody dupilumab, which blocks IL-4 and IL-13, reduced total IgE levels in clinical trials, thus also indicating that de novo class switching may occur in humans [71,72,73,74]. However, when interpreting sequencing data several aspects need to be considered carefully. Firstly, next generation sequencing only analyzes heavy and not light chain sequences. Furthermore, so far it is not possible to investigate the allergen-specificity through the use of this technique. Although IgE+ memory cells have been observed in the blood of allergic subjects [46], their switch origin and their contribution to IgE production has not been elucidated. Additionally, the relative contribution of direct versus sequential class switching to the IgE pool is not yet fully understood either.

In summary, continuous IgE production, most likely by long-lived plasma cells as well as by memory cells rapidly responding to allergen by IgE production, seem to contribute to the maintenance of allergen-specific IgE levels in allergic patients despite the short half-life of IgE.

## 3. Tracing IgE Producing Cells

Another open question is where the major sites of IgE production are in humans. The majority of studies investigating mechanisms of human IgE production have used peripheral blood mononuclear cells (PBMCs) [24,75,76,77] or tonsil derived B cells [78,79]. Though in vitro cultures using isolated PBMCs have provided important insights on the role of specific T cell help and IL-4 in IgE production [77,80,81,82,83], these studies are not helpful for understanding allergen-specific IgE production because IL-4 stimulation induces class-switching in any IgM+ B cells but does not influence allergen-specific IgE production [77]. Since the demonstration that one can isolate allergen-specific IgE Fabs from the peripheral blood of allergic patients by combinatorial cloning [84], it is clear that cells producing allergen-specific IgE occur in the blood of allergic patients, but the nature of these cells needs to be characterized in more detail [25]. In order to claim that one has identified a major site of IgE production in allergic patients, one would need to demonstrate that the IgE-producing cells in blood are derived from this site and/or that the majority of IgE is synthesized at this particular site [25,85].

### 3.1. Identification of IgE Producing Cells by IgE Staining Techniques

The assumption that IgE-producing cells are mainly localized in the bone marrow and other human lymphoid compartments is difficult to prove because these tissues are quite difficult to access in humans. It seems to be more convenient to work with human peripheral blood; however, the amount of IgE-producing cells in human blood is minimal and there are numerous cell types that carry IgE on their surface but do not produce it. In this section, we summarize current approaches for the identification of IgE-producing cells.

#### 3.1.1. ELISpot

One possible method for detecting IgE-producing cells in the blood is the ELISpot assay. This method is more effective for plasmablasts than for the determination of memory B cells, as the latter do not secrete antibodies. In order to obtain antibody-secreting cells from B cells, stimulation with CD40L and IL-4 or allergen is usually performed [86,87]. However, as pointed out, this stimulation does not amplify existing allergen-specific IgE production but leads to de novo class switching of IgM+ BCR bearing cells of unknown specificity into IgE producing cells [77,86,87].

#### 3.1.2. Flow Cytometry 

The most commonly used method for detecting and isolating putative IgE-producing B-lymphocytes is flow cytometry, which is based on the determination of surface markers of target cells. There are several approaches for IgE+ B lymphocyte detection. For the step-by-step determination of the total subpopulation of IgE+ B lymphocytes from the PBMC pool, first, non-relevant cells (e.g., T cells, monocytes) are excluded by cells expressing the following markers: CD3, CD14, CD16, CD235a, and CD123. Consequently, IgE+ B lymphocytes are identified by positive staining for CD19 as well IgE. However, this approach does not exclusively identify IgE+ memory cells as IgE can be present on the B cell surface not only in the form of the IgE B cell receptor (BCR) but also bound to its low affinity receptor CD23 [88]. This fact needs to be considered carefully, as it may lead to overestimation of the number of IgE+ B cells.

In addition to the above-stated panel, antibodies to CD23 can be added, which should help to exclude false-positive events from the gate of IgE+ cells. However, by excluding all CD23+ lymphocytes, one may potentially remove the subset of IgE-producing cells that express CD23 at the same time.

Compared to the previous approach a more successful strategy may be enrichment of B lymphocytes by using magnetic separation or RosetteSep prior to antibody staining [89,90]. This has two main advantages: Firstly, the number of cells required for measurement is strongly decreased. Secondly, antibodies targeting further B cell-related surface markers may be implemented as no markers for exclusion of irrelevant cells are necessary.

Antigen-specific labeling of B lymphocytes is in general a successful method for isolating B cells responding to a particular antigen [91]. A crucial determinant in this approach is the production of antigen with a high level of fluorescence. A high level of fluorescence can be achieved using tetrameric technology, by which a complex is preformed from fluorescently labeled avidin bound to four biotinylated antigen molecules [92].

Taking into consideration the low percentage of target cells compared to background levels, it may be preferable to use several antigen conjugates labeled with different fluorescent labels in order to show reproducible target cell isolation. 

For a more reliable isolation of IgE+ B lymphocytes, this strategy can be combined with additional staining for other surface Igs (IgM, IgD, IgG, IgA) [93,94]. Jiménez-Saiz et al. [86] applied this strategy for the isolation of IgE+ memory cells from human blood. They stained B cells enriched from PBMCs by magnetic separation with anti-IgM, anti-IgD, anti-IgG, anti-IgA, and anti-IgE antibodies. Cells were analyzed and IgE+ subpopulations were isolated by using flow cytometry. The purity of the IgE+ subpopulation was then confirmed by single cell sequencing and revealed that only 0.0019% of total B cells were putative IgE+ memory B cells [86] and that these cells were rarer than previously reported [46,89]. This very low percentage is rather challenging since it is even less than the frequency of residual tumor populations, which is observed in minimal residual disease [95]. 

Furthermore, circulating IgE+ B lymphocytes can be divided into plasmablasts and memory cells based on antibodies against CD27, CD38, and CD138 [25]. Using this approach, Heeringa et al. identified the following IgE+ subsets from donor blood samples: IgE+CD27− memory B cells (CD19+CD21+CD38dimIgD-IgM-IgE+CD27-), IgE+CD27+ memory B cells (CD19+CD21+CD38dimIgD-IgM-IgE+CD27+), and IgE+ plasmablasts (CD19+CD38highCD27+IgM-IgD-IgE+) [89]. Alternatively, plasmablasts can also be identified using cellular affinity matrix technology in negatively enriched B cells [24]. 

To summarize, the major obstacles in any attempt to characterize IgE-producing plasma cells and potentially IgE memory B cells in the blood of allergic patients are the small numbers of allergen-specific lymphocytes [24,86] and the difficulty in clearly identifying them by flow cytometry [28]. 

**Figure 2:**
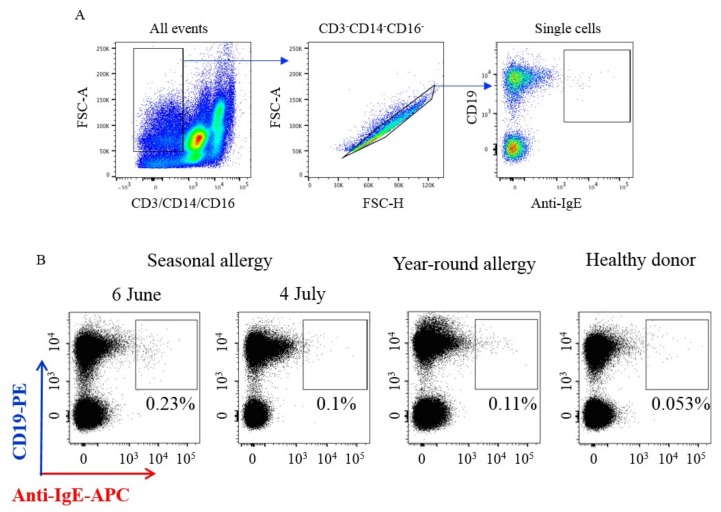
Isolation of IgE+ B lymphocytes from PBMC of donors with different type of allergy. (**A**) Gating strategy for detection of IgE+ B lymphocyte. B lymphocyte subset were gated as CD3-CD14-CD16- (FSC area/CD3, CD14, CD16), single cells (FSC area/FSC height), CD19+, and then detection of IgE+ B cells was based on surface IgE expression. (**B**) Example of different percentage of IgE+ B cells in donor samples with different types of allergy. The highest percentage of IgE+ cells were detected in a donor sample with seasonal allergy shortly after the pollen season (6th June) and they disappeared a month later (4th July). As a control, we used blood samples from healthy donors, as well as donors with year-round allergies.

In an attempt to overcome this obstacle, we have recently developed an approach where cells were stained for CD19 in combination with a fluorescently labelled monoclonal anti-IgE antibody, which discriminates membrane-bound IgE from receptor-bound IgE in the blood of birch pollen allergic patients (Figure 2). 

To detect IgE-producing B lymphocytes of patients with seasonal birch pollen allergy, we isolated PBMCs and stained them with a cocktail of antibodies CD19-PE, CD3-FITC, CD14-FITC, CD16-FITC, and anti-IgE-APC. Figure 2 shows that IgE-producing B-lymphocytes accounted for 0.23% of subpopulations of CD19+ cells after the birch pollen season (Figure 2, 6th June) and after one more month (Figure 2, 4th July) this subpopulation disappeared. This would suggest that IgE-producing cells appear in the peripheral blood after allergen exposure. In fact, they seem to be present in allergic patients with a perennial allergy (Figure 2, Year-around allergy) and are absent in a healthy non-allergic donor blood (Figure 2, Healthy donor). 

### 3.2. Sites of IgE Production

**Figure 3:**
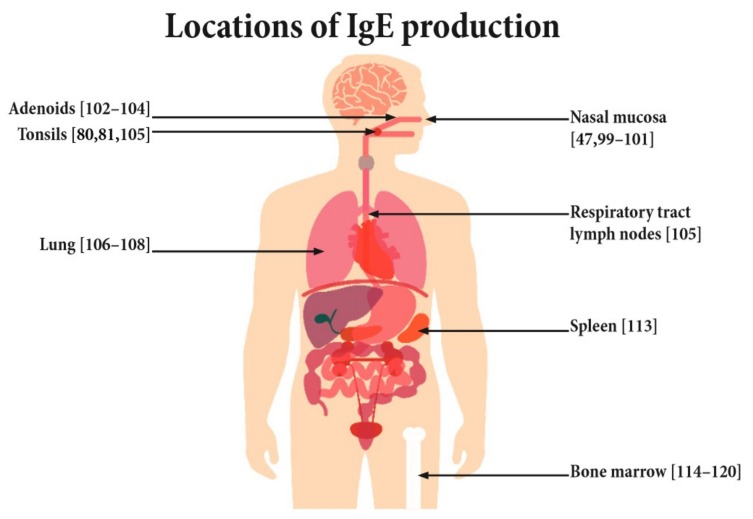
Potential sites of IgE production.

Though both IgE-producing plasma cells and IgE+ memory cells have been observed in the blood of allergic patients [24,46,86,89], they are scarce and produce only around 0.2% of the IgE present in the serum [25]. Thus, the majority of IgE producing cells and IgE memory are thought to reside elsewhere [85]. Human B memory cells, for example, in response to vaccinations, have been shown to reside not only in the bone marrow but also in other different lymphatic organs such as the spleen and tonsils [91,96,97,98]. Likewise, IgE production has been suggested to occur locally at several different sites throughout the body (Figure 3). For example, at the nasal mucosa, the site of first contact for airborne allergens, increased numbers of IgE-positive B and plasma cells have been observed [99]. The presence of local IgE synthesis by the detection of ε germline and ε circle transcripts have been shown in nasal mucosa biopsies from allergic patients after ex vivo challenge with the respective allergen [47,100,101,102]. In addition, there are various other lymphatic tissues of the upper respiratory tract such as adenoids and tonsils that are potential sites of antigen encounter after uptake of the antigen by the nasal mucosa. These lymphatic tissues have also been shown to harbor IgE+ cells [102,103] and the production of IgE has been shown [78,79,104,105]. Similarly, IgE transcripts and antibodies have been detected in the sputum and lungs of allergic and asthmatic patients [106,107,108]. However, it should be noted that blood-derived cells (which includes IgE producing cells) also contribute to the cellular population of these lymphatic and respiratory tissues, and therefore it is difficult to say if these tissues really are predominant sites for IgE production.

As sites known to house large number of memory cells, both the spleen and bone marrow are potential sites of interest [91,97,109,110,111]. However, the contribution of the spleen in maintaining even the IgG repertoire and memory is still discussed. Although vaccinia virus-specific memory B cells are present in the spleen of patient’s even decades after smallpox has been eradicated [112], splenectomized individuals mount comparable anti-tetanus toxoid IgG levels upon revaccination to normal healthy patients [91]. Even less is known about the presence of IgE production in the spleen. Only one report observed the presence of IgE production in the spleen of an asthmatic patient who tragically died from an asthma attack [113]. With regard to the presence of IgE production in bone marrow there are several observations from patients receiving allogenic donations, which show the transmission of allergy after bone marrow transplantation [114,115,116,117,118,119,120]. However, results are somewhat ambiguous. Some reports observe matching sensitization profiles between donors and recipients [114,115,116,117] whilst others show that recipients may not acquire all the allergies of the donor [118,120] or may even acquire additional de novo sensitizations [119]. 

The presence of IgE production has so far been shown at various lymphatic sites; however, the relative contribution of each of these areas to the continuous and de novo production in allergic responses remains unanswered. One may speculate that lymphatic tissues at the sites of allergen exposure (e.g., nasal mucosa, gut mucosa) contain IgE+ memory cells, which can be activated, whereas plasma cells in bone marrow may be responsible for the continuous long-lived IgE production. Nevertheless, further investigations are needed to identify the sites of IgE production in allergic patients.

## 4. Role of IgE in Mediating Immediate Allergic Symptoms and T Cell-Mediated Allergic Inflammation

IgE plays an important role in propagating allergic responses both in the early and late phases of allergic immune responses. In the early phase, IgE is central for mast and basophil degranulation upon allergen contact; however, the timely interplay between increases in IgE levels in the blood upon allergen challenge and potential rises in effector cell sensitivity are not yet fully understood. In the late phase, IgE-bearing B cells contribute to increased allergen-specific T cell response via internalization and presentation of IgE-allergen complexes via CD23 in a process called IgE-facilitated allergen presentation. 

### 4.1. Connecting IgE Production to Clinical Effect—How Circulating IgE Influences Mast Cell and Basophil Sensitivity

The major players propagating the early symptoms experienced by allergic individuals in immediate hypersensitivity reactions are basophils and mast cells. Both of these cells utilize IgE to mediate their allergic responses, and thus understanding the kinetics of how circulating IgE influences the reactivity profile of these cells is crucial to treating the disease. Basophils and mast cells are similar in that they are both granulocytes, both are derived from hematopoietic stem cells, and both contain pre-formed intracellular granules that are rapidly exocytosed from the cell membrane upon FcεRI cross linking in response to IgE allergen recognition [121]. Despite their similarities there are some key differences to consider. Firstly, mast cells are long-lived tissue-resident cells, surviving for months. Basophils on the other hand circulate in the blood and survive only for a few days. Basophils terminally differentiate in the bone marrow whereas mast cells do so in the tissues. Additionally, basophils do not usually proliferate after maturation whereas mast cells can in order to self-renew [122]. 

Due to the high affinity of FcεRI for IgE, receptor bound IgE—in contrast to free IgE—has a relatively long half-life of 2–3 weeks [123]. Additionally, circulating IgE has been shown to have a positive effect on the stability and expression of FcεRI expression on mast cells and basophils by preventing receptor internalization and degradation [124]. These two mechanisms of slow off rate and increased FcεRI expression and stability mean that once IgE is bound to its receptor it remains bound most likely for the lifespan of the host cell. Thus, it would be reasonable to presume that the specificity of IgE in the circulation will reflect the reactivity profile of mast cells and basophils. This has indeed been shown to be the case in that specific serum IgE levels correlate quite well with skin prick test results in adults [125,126]. 

**Figure 4:**
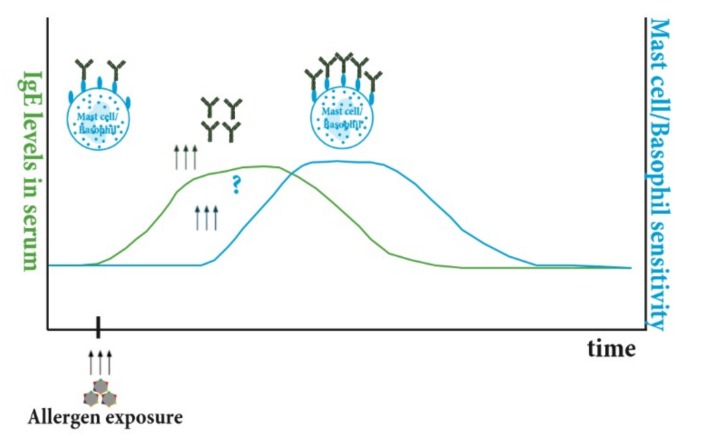
Timely interplay of rises in IgE levels and mast cell and basophil sensitivity. Upon allergen exposure there is a rise in allergen-specific IgE levels (green line) followed by an increase in basophil and mast cell sensitivity (blue line).

Nasal allergen exposure in pre-sensitized individuals leads to a rise in allergen specific IgE with peak serum levels occurring 4–6 weeks after exposure [37,127,128]. Taking into consideration the differences in life span of basophils and mast cells it would be logical to assume that basophils would be the first to reflect this change in the IgE repertoire followed later by mast cells (Figure 4). So far there is no study directly addressing this question; however, conclusions can be drawn from allergen challenge or therapeutic studies removing IgE from the circulation. Upon intranasal allergen challenge, no change in basophil sensitivity was observed within the observation period of 3 weeks after peak allergen-specific IgE increase [128]. However, in an extracorporeal IgE-specific immuneapheresis study by Lupinek et al., a reduction in basophil sensitivity was observed 8 weeks after start of the treatment [13]. Similarly, basophil sensitivity continuously dropped after neutralization of IgE with the anti-IgE antibody omalizumab [129,130,131,132]. IgE surface levels on basophils were undetectable 4 weeks after omalizumab treatment but rose significantly 8 weeks after cessation of treatment [133]. Thus, these data indicate that there is a time window of at least 4–8 weeks for changes in IgE levels to be reflected in changes of basophil sensitivity. With regards to changes in skin prick tests (SPT), which act as a surrogate of mast cell sensitivity, the seasonal increase in SPT sensitivity observed several weeks after the start of the study in birch allergic control patients was absent in patients undergoing IgE immunoapheresis [13]. Furthermore, a reduction in SPT reactivity was also observed in omalizumab-treated patients within 3 months of treatment [131,134]. This indicates that it takes longer for mast cells than basophils to adapt to changes in IgE levels, as expected, due to their longer life-span. However, a detailed study addressing the timely interplay has not been performed yet and would be needed for a better understanding of how the kinetics of the IgE response transform into clinical sensitivity. 

### 4.2. Importance of IgE-Facilitatated Allergen Presentation Mediated by CD23 and Rules Guiding this Process

The activation of allergen-specific T cells by APCs plays an important role in the development of an allergic reaction, especially in mediating late phase reactions [135]. In fact, T cell activation increases the levels of Th2 cytokines such as IL-4, IL-13, and IL-5, which are important for eosinophil recruitment into the target tissues of allergic inflammation and leads to subsequent tissue damage and remodeling. The uptake of IgE-allergen complexes by CD23 was firstly described nearly 30 years ago [136] and the process was termed IgE-facilitated allergen presentation (IgE-FAP). CD23 is mainly expressed on the surface of resting naïve IgD+ B cells [88]. Upon binding of IgE-allergen complexes to CD23, these complexes are endocytosed and processed, leading to the loading of allergen-derived peptides on MHC II, which can be recognized by specific T cells [137]. Alternatively, CD23 bearing primary B cells may also transfer IgE-allergen complexes to dendritic cells for processing of the allergen and presentation of allergen-derived peptides to T cells [138,139]. CD23 may also play an important role in the transcytosis of IgE and IgE-antigen complexes across human intestinal [140] and respiratory epithelial cells [141], as well as in transporting IgE-antigen complexes to B cell follicles in mice [142]. IgE-FAP is a very efficient process of inducing T cell activation, as 100–1000 fold lower amounts of allergen complexed with specific IgE than allergen alone were needed to trigger T cell activation in vitro [6,143]. Moreover, allergen-specific T cell activation by CD23-mediated FAP is accompanied by the release of pro-inflammatory cytokines [6]. The blocking of CD23 with a specific anti-CD23 antibody, lumiliximab, in allergen-stimulated PBMCs reduced allergen-specific T cell activation by 50%, highlighting the role of IgE-FAP in allergen presentation [144]. Its importance is underlined by the fact that IgG blocking antibodies, which are induced upon immunotherapy, inhibit IgE-FAP, thus reducing specific T cell proliferation and the release of pro-inflammatory cytokines [145,146,147,148,149,150]. Besides its role in mediating IgE-FAP, CD23 is also known to regulate serum IgE levels in murine models by capturing IgE by CD23-expressing cells [151,152].

**Figure 5:**
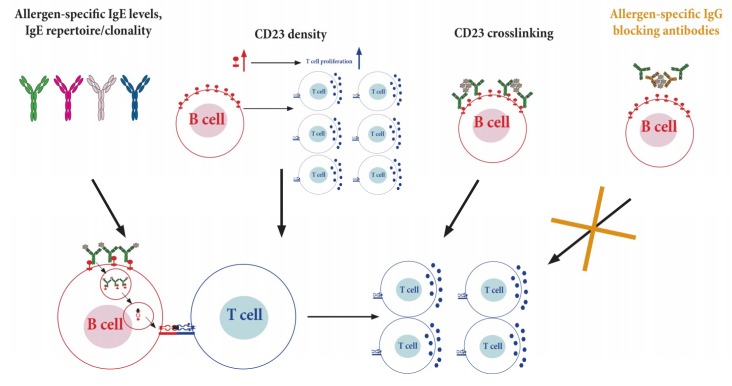
Factors affecting the extent of CD23-mediated facilitated allergen presentation (FAP) and subsequent T cell activation. Binding of IgE-allergen-complexes to CD23 present on B cells (bottom section, red cell) leads to endocytosis of these complexes followed by processing and loading of allergen-derived peptides on MHCII, which can be recognized by specific T cells (blue cells). This process, called IgE FAP, enhances activation and proliferation of T cells. Factors involved in controlling IgE-FAP include allergen-specific IgE levels, IgE repertoire/clonality, CD23 density, the extent of IgE crosslinking, and allergen-specific blocking IgG antibodies.

It is therefore important to understand the modes of how IgE-allergen complexes bind to CD23 [153] and the factors controlling the extent of CD23-mediated IgE-FAP (Figure 5). Allergen-specific IgE levels and the complexity of the IgE repertoire, with regards to their clonality and affinity for an allergen, determine the formation of IgE-allergen complexes, and therefore affect the activation of allergen-specific T cells [154]. Recently, CD23 density on B cells has been described to correlate with IgE levels in the serum and is associated with the extent of allergen-specific T cell activation [88]. Additionally, other factors might be involved in controlling IgE-FAP such as the extent of CD23 crosslinking. Further studies analyzing the afore mentioned components of CD23 would help to shed light on the different elements affecting IgE-FAP. 

## 5. Targeting of IgE and IgE+ Cells

As IgE is central to mediating symptoms of allergic diseases, it represents an important and attractive target for developing novel therapeutics (Appendix A Table A1). There are currently two different approaches: targeting of the antibody itself or interfering with the activation of IgE receptor-bearing effector cells. The second is the elimination of IgE-producing cells to inhibit IgE production at its origin. 

### 5.1. Targeting IgE and Interference with Activation of Effector Cells 

The heavy chain of IgE is composed of four constant Cε domains. The binding site for IgE to its high and low affinity receptors has been mapped to the Cε3 domain [155,156]. Thus, the first generation of therapeutic anti-IgE antibodies were designed to bind selectively to this domain to reduce free IgE levels and to inhibit binding of IgE to its receptor thereby leading to strong reduction in activation of mast and basophils upon allergen contact [157,158,159]. Administration of omalizumab, the first licensed humanized monoclonal IgG_1_ antibody directed towards IgE, has been shown to reduce free IgE levels by 99% within 2 h of administration, and reduces human basophil responsiveness within 3 months [129,160]. It may also inhibit IgE synthesis in B cells in vitro [161]. It is successfully used for treatment of severe asthma as well as urticaria [17,162]. Recently, a biosimilar antibody to omalizumab was developed by Shanghai Biomabs Pharmaceutical Co., Ltd. [163] and is currently being tested in 400 asthmatic patients in a multicenter phase III trial (NCT03468790), which is expected to be finished by December 2019. Ligelizumab (QGE031) is also targeting the Cε3 region but with a 50-fold greater affinity than omalizumab in vitro. However, in a clinical study, asthmatic patients receiving ligelizumab performed only slightly better than omalizumab-treated patients [164]. Phase III clinical trials with ligelizumab in chronic spontaneous urticaria are still ongoing (NCT03580369, NCT03580356). MEDI4121 is another humanized IgG_1_ antibody for neutralization of free IgE domain and binds selectively the Cε3 and Cε4 region of IgE [165]. It bears a 106-fold greater affinity for IgE as compared to omalizumab. Consequently, administration in a pharmacokinetic study of MEDI4121 in humans led to a rapid decrease of IgE [166]. However, MEDI4121 is eliminated quickly in vivo resulting in the quick recovery of free IgE levels back to baseline. Thus, despite having a higher affinity, the antibody would need to be administered at short intervals to maintain IgE suppression and currently no further studies have been performed. In addition, macromolecular inhibitors, so called designed ankyrin repeat proteins (DARPins), have been developed that do not interact with free IgE but actively promote the dissociation of receptor-bound IgE from FcεRI [167]. DARPins have been shown to efficiently prevent passive cutaneous sensitization in mice [168]. Recently, the research group of Alexander Eggel has developed a bispecific DARPin co-ligating the inhibitory FcγRIIB with FcεRI bound IgE on effector cells. This specific targeting strategy resulted in reduced allergen-induced effector cell degranulation as well as inhibition of systemic anaphylaxis in vivo [169]. The approach of inducing auto-antibodies to human IgE receptor-binding sites by peptide vaccination has only been tried in rodent models [170,171] and so far has not been brought forward to clinical testing. As an alternative approach, especially in patients who are not suited for omalizumab therapy due to excessive IgE levels, IgE may also be removed from the blood using extracorporal immunoapheresis. Here, two studies showed that immunoadorption using anti-IgE antibodies reduced peripheral IgE levels by around 90% in patients with IgE levels up to 10,000 kU/L [13,20]. Immunoadsorption led to improvement of clinical symptoms both in atopic dermatitis and in allergic asthma. Thus, it may additionally be a valuable pre-treatment for patients with very high IgE levels to enable them to start omalizumab therapy.

Another strategy aims at reducing the number of effector cells. In mouse models it has been shown that amelioration of allergic disease can be achieved by targeting mast cells and basophils with anti-FcεRIa Fab coated micelles loaded with celastrol [21]. The latter is a quinone methide triterpene derived from *Tripterygium wilfordii* and capable of potentiating TNF-induced apoptosis [172]. CTLA4Fcε, a Th2 modulatory component, has so far only been tested in vitro [22]. CTLA4Fcε is a recombinant fusion protein of the ectodomain of the immunoregulatory molecules cytotoxic T lymphocyte antigen 4 (CTLA-4) with a fragment of IgE heavy chain constant region and thus binds to IgE receptors as well as CD80 and CD86. Due to these properties it is thought to reduce both IgE production via soluble CD23 as well as lymphocyte proliferation.

### 5.2. Therapeutic Targeting of IgE-Producing Cells

Several approaches have been made to eliminate IgE-producing cells. For example, quilizumab is directed towards the M1 domain of the membrane-bound IgE B cell receptor and was shown to reduce IgE levels as well as numbers of IgE-producing plasma cells in a murine model [173]. Despite a reduction of serum IgE in humans by 25–40% [23,174], a large clinical trial with more than 500 patients suffering from allergic asthma uncontrolled by standard therapy showed no reduction of asthma exacerbations within the 36 weeks of treatment [23]. However, the IgE levels, especially in the treatment group receiving the highest dose of quilizumab, showed a continuous decline during the 48 weeks of safety follow up. Similar results were obtained in a study where chronic spontaneous urticaria was treated with quilizumab [175]. Taking into account the longevity of plasma cells that cannot be destroyed by this approach due to the absence of a B cell receptor, it is conceivable that the treatment effect may have been underestimated due to the relative shortness of the treatment and observation period. Alternatively, a bispecific IgE-CD3 antibody has been developed with the aim of destroying IgE-bearing B cells by directing the cytotoxic activity of T cells to them [176]. It is directed towards epitopes of IgE, which are inaccessible when IgE is bound to its Fc receptors [177]. Initial in vitro experiments have shown that this non-anaphylactogenic antibody is capable of inducing lysis of IgE+ membrane B cells by cytotoxic T cells [176]; however, whether it will also be successful in treatment of allergic patients has not been studied. Other approaches aim at targeting or co-targeting of Fcγ receptors for the elimination of IgE+ B cells. In this respect, mutations of the above mentioned anti-IgE antibody MEDI4121 initially developed for binding to free serum IgE have been selected with improved binding to FcγRIIIa, a receptor involved in antibody-dependent cellular toxicity [178]. Another Fcγ receptor, namely FcγRIIb, is also involved in down regulation of BCR signaling and is thus targeted by XmAb8915, an antibody that co-engages FcγRIIb and the IgE B cell receptor [179]. It was shown to successfully reduce IgE production by PBMCs in vitro and to specifically reduce IgE production in SCID mice engrafted with human PBMCs. In a very recent approach, off-springs of mice vaccinated with anti-IgE during pregnancy showed suppressed IgE levels in response to antigen challenge [180]. This suggests that treatment with anti-IgE antibody during pregnancy could prevent allergic sensitization.

## 6. Conclusions

Though IgE is the least abundant class of immunoglobulins with an extremely short half-life it plays a central role in allergic disease. Allergen-specific IgE production in allergic patients seems to consist of two modes: a continuous mode, which maintains IgE levels even in the absence of an allergen stimulus, and a reactionary mode, where increases of IgE production occur after exogenous allergen stimulus. Neither the precise sites nor the nature of the IgE-producing cells involved in these two modes of IgE production in allergic patients are known. Another as yet unanswered matter is the timely interplay between rises in IgE production occurring in response to allergen exposure and the loading of effector cells capable of binding IgE via their high and low affinity receptors. Due to fundamental differences between allergic patients and experimental animal models for allergy, research in patients will be required to address the open research questions regarding IgE-producing cells, mechanisms, and sites of IgE production, as well as the loading of IgE to effectors cells, which is responsible for allergic inflammation. Clinical experience with therapeutic strategies depleting IgE and preventing IgE binding to its receptors indicates that the inactivation IgE-mediated effector cell activation is not harmful. The elimination of IgE-producing cells may therefore represent a safe therapeutic strategy, which may lead to a cure of allergy but will require the identification and characterization of IgE-producing cells in allergic patients.

## Figures and Tables

**Table 1:** Comparison of allergy in humans and murine models.

	Mice	Humans
Genetic background	Inbred	Outbred
Percentage lymphocytes of total leukocytes	75–90% [39]	30–50% [40]
IgG subclasses	IgG_1_, IgG_2_, and IgG_3_	IgG_1_, IgG_2_, IgG_3_, and IgG_4_
IgE receptors on eosinophils	No FcεRI [41]	FcεRI [42]
Access to tissue for analysis	All tissues available	Limited access—mainly blood
Asthma development	Induced by sensitization, sometimes Th1-like	Induced by natural allergen exposure, mostly Th2-like
Allergy	Induced by sensitization [43,44,45]	Spontaneous by natural allergen exposure
IgE epitopes of respiratory allergens	Mainly sequential	Mainly conformational
T cell epitopes of respiratory allergens	Dominating T cell epitopes	High diversity
Cytokines required for IgE class switch	IL-4 [31]	IL-4, IL-13 [32]
Mechanisms of class-switch to IgE	Mainly sequential [33]	Evidence for sequential [46,47,48,49,50,51,52] and non-sequential [53]
Rise in Ig subtype in response to allergen challenge	IgE, IgG_1_	IgE, IgG_4_

## Appendix A

**Table A1:** (**A**) Therapeutic approaches targeting effector IgE^+^ and IgE producing cells. Randomised Control Trial (RCT), Adverse Event (AE). (**B**) Therapeutic approaches targeting effector IgE^+^ and IgE producing cells. Randomised Control Trial (RCT), Adverse Event (AE).

**A**	**Approach**	**Biological Effect**	**Study Design**	**Disease Target**	**Subjects**	**Length of Observation**	**Outcome**	**Current Phase**	**References**
**Strategies targeting IgE**	Immuno adsorption	Removal of circulating IgE or total Ig through Plasmapheresis	RCT	Allergic Asthma	N = 15	16 weeks	↓Total IgE ↓ basophil activation	Pre-Clinical	[13]
			RCT	Atopic Dermatitis	N = 50	8 weeks	Less AE in IgE group vs pan Ig	Pre-Clinical	[20]
	Omalizumab	Monoclonal antibody against Fc portion of IgE—prevents receptor binding	RCT	Allergic Asthma	N = 317	20 weeks	↓IgE ↓Steroid use	Marketed	[17]
	CMAB007	Biosimilar to Omalizumab developed by China	RCT	Allergic Asthma	N = 400	24 weeks	Not yet completed	Phase III NCT03468790	[163]
	DARPins	Ankyrin repeat domains that affect stability and function of target protein	In vitro study	Allergy	Isolated basophils	N/A	Removal of IgE from basophils + ↓ basophil activation	Pre-Clinical	[168]
	MEDI4212	Monoclonal antibody against Fc portion of IgE—prevents receptor binding	RCT	Allergy/Atopy	N = 86	12 weeks	Greater ↓total IgE vs Omalizumab worse half life	Phase I NCT01544348	[166]
	MEDI4212 Variant	Monoclonal antibody against Fc portion of IgE and Fc potion of monoclonal antibody binds to inhibitory receptor FcγRIIIa on B-cells	In vitro study	Allergy	Cell lines and human B cells	N/A	Elimination of IgE expressing B cells	Pre-Clinical	[178]
	IgE Peptide Vaccine	Induction of autoantibodies against Fc region of IgE	In vitro study	Allergy	FcεRI–ELISA	N/A	Autoantibodies block IgE binding to FcεRI	Pre-Clinical	[171]
	QGE031 (Ligelizumab)	Monoclonal antibody against Fc portion of IgE—prevents receptor binding	RCT	Allergic Asthma	N = 37	10 weeks	QGE031 > Omalizumab	Phase II NCT01703312	[164]
**B**	**Approaches**	**Biological Effect**	**Study Design**	**Disease Target**	**Subjects**	**Length of Observation**	**Outcome**	**Current Trail Phase**	**References**
**Strategies targeting IgE production or effector cells**	Quilizumab	Monoclonal antibody targeting M1-prime segment of membrane bound IgE expressed on IgE switched B cells leading to cell depletion	RCT	Allergic Asthma	N = 578	36 weeks with 48 week safety follow-up	Acceptable safety and reduced serum IgE but no clinically meaningful benefit in clinical outcome parameters	Phase II NCT01582503	[23]
	DARPins	Ankyrin repeat domains that affect stability and function of target protein	In vitro study	Allergy	Human basophils	N/A	Targets FcγIIB and inhibits basophil degranulation	Pre-Clinical	[169]
	Bsc-IgE/CD3 Construct	Monoclonal antibody binding to cells with membrane bound IgE and targets T-cell cytotoxic activity towards them	In vitro study	Allergy	Cells isolated from allergic human donors	N/A	Bsc-IgE/CD3 effective at eliminating IgE+ B cells without inducing degranulation of mast cells	Pre-Clinical	[176]
	Anti-FcεRI Fab-conjugated celastrol-loaded micelles	Fusion with cell membrane and induction of apoptosis of FcεRI expressing cells	In vitro study	Allergy	Mast cell line	N/A	Efficient induction of apoptosis of mast cells and reduction of allergic inflammation in mouse model	Pre-Clinical	[21]
	CTLA4Fcε Fusion Protein	Binds FcεRI and CD23, prevents CD23 cleavage, and blocks CD80/CD86 costimulation	In vitro study	Allergy	Cell line and human PBMC samples	N/A	Reduces sCD23 and lymphocyte proliferation	Pre-Clinical	[22]
	Maternal Anti-IgE Vaccination	IgG anti IgE antibodies transferred from mother to fetus and prevent onset of allergy by targeting IgE memory B cells	In vivo mouse study	Allergy	N/A	9 weeks after birth	Reduced IgE levels in mouse offspring	Pre-Clinical	[180]

## Abbreviations

APC	Antigen Presenting Cell
BCR	B Cell Receptor
CD23	Low Affinity Receptor for IgE
FcεRI	High Affinity Receptor for IgE
IgE	Immunoglobulin E
MHC	Major Histocompatibility Complex
PBMC	Peripheral Blood Mononuclear Cell
